# The squiggle tail (*squig*) mutation in mice is associated with a deletion in the mesenchyme homeobox 1 (*Meox1*) gene

**DOI:** 10.1186/s13104-022-06192-z

**Published:** 2022-09-23

**Authors:** Jon P. Girard, Jacqueline F. Tomasiello, Juan I. Samuel-Constanzo, Nia Montero, Angelina M. Kendra, Thomas R. King

**Affiliations:** Department of Biomolecular Sciences, Central CT State University, 1615 Stanley Street, New Britain, CT 06053 USA

**Keywords:** Positional cloning, Tail variant, Klippel-Feil syndrome 2, Deletion mutation

## Abstract

**Objective:**

We have taken a positional approach to assign the spontaneous squiggle tail (*squig*) mutation in mice to a specific gene defect.

**Results:**

A large panel of backcross mice was produced and characterized to map *squig* to high genetic resolution on mouse Chromosome (Chr) 11. Two overlapping candidate genes that co-localized with *squig* (*Meox1*, for mesenchyme homeobox 1; and *Gm11551*, which encodes a lncRNA located entirely within the first intron of *Meox1*) were fully sequenced to discover any *squig*-specific defects. This analysis revealed a 3195 bp deletion that includes all of *Meox1*, *Exon 1* but does not disrupt *Gm11551*. We recommend that the *squig* mutation be renamed *Meox1*^*squig*^, and suggest that this variant may offer an appropriate animal model for Klippel-Feil syndrome 2 (KFS2) in humans.

**Supplementary Information:**

The online version contains supplementary material available at 10.1186/s13104-022-06192-z.

## Introduction

The recessive squiggle tail mutation (abbreviated *squig*) arose spontaneously in the BALB/cJ inbred mouse strain at The Jackson Laboratory (Bar Harbor, ME, USA) in 2013, and has been maintained on a segregating, coisogenic background since that time. Mice homozygous for *squig* display a shortened and very curly tail and are frequently smaller than their non-mutant littermates (see Additional file 1: Figure S1). In 2016, Karst et al. [1] mapped *squig* to mouse Chromosome (Chr) 11, based on the analysis of single nucleotide polymorphisms (SNPs) among a small set of F_2_ homozygotes, but the genetic resolution achieved was not sufficient to suggest any causative gene.

As a basis for assigning the squiggle tail phenotype to a specific genetic cause—which would facilitate the further analysis of this interesting variant and help to identify an orthologous human disorder—here we have fine-mapped *squig* with respect to various microsatellite and SNP markers on mouse Chr 11. This analysis identified a small set of co-localizing candidate genes, and we now suggest that one of these, *Meox1* (for mesenchyme homeobox 1), harbors the *squig* defect.

## Main text

### Methods

#### Mice

Mice from the standard inbred strains C57BL/6 J (JAX stock #000664) and BALB/cJ (JAX stock #000651), and co-isogenic BALB/cJ-*squig*/GrsrJ mice (JAX stock #026620) were obtained from The Jackson Laboratory (Bar Harbor, ME, USA). The *squig* mutation was maintained at CCSU by crossing heterozygotes with mutant homozygotes. Mutants were reliably identified (with at least 99.3% penetrance in our colony) by their short and curly tails that are apparent from birth (see Additional file 1: Figure S1). At the end of the study, mice were killed by cervical dislocation or by use of CO_2_ gas added to a chamber (typically their home cage) using a compressed gas cylinder fitted with a flow meter adjusted to displace only 30–70% of the chamber volume per minute (consistent with the recommendations of the *AVMA Guidelines for the Euthanasia of Animals, 2020 Edition*). Only the P.I. (TRK) who was trained at The Jackson Laboratory (Bar Harbor, ME) and has over 30 years of experience, performed euthanasia.

#### DNA isolation and marker typing

Genomic DNA was isolated from 2 mm tail-tip biopsies taken from two- to three-week-old mice using NucleoSpin^®^ Tissue kits (Macherey–Nagel, Düren, Germany; distributed by Clontech Laboratories, Inc., Mountain View, CA, USA), as directed. DNA samples from standard inbred and mutant strains that we do not routinely maintain in our colony were purchased from The Jackson Laboratory’s Mouse DNA Resource.

The polymerase chain reaction (PCR) was performed in 13 µl reactions using the Titanium^®^ PCR kit from Clontech Laboratories, as directed. Oligonucleotide primers for PCR were designed and synthesized by Integrated DNA Technologies, Inc. (Coralville, IA, USA), based on sequence information available online [2, 3]. To score PCR product sizes for dimorphic microsatellite markers, reactions plus 3 µl loading buffer were electrophoresed through 3.5% NuSieve^®^ agarose (Lonza, Rockland, ME, USA) gels. Gels were stained with ethidium bromide and photographed under ultraviolet light. In addition to eight standard microsatellite markers [4] on Chr 11, eight DNA markers based on single nucleotide polymorphisms previously reported to differ between strains BALB/cJ and C57BL/6 J [2, 3] were scored. These markers (herein designated *SNP#*) are described in detail in Additional file 2: Table S1 and Additional file 3: Table S2.

#### Sequence analysis

For DNA sequence analysis, about 1.5 µg of individual PCR amplimers were purified and concentrated into a 30 µl volume using NucleoSpin^®^ PCR Clean-up kits, and then shipped to the Keck Foundation Resource Laboratory at Yale University (New Haven, CT, USA) for primer-extension analysis.

#### ***A “3-primer” test for detecting*** Meox1^squig^***alleles***

To rapidly determine genotypes at the *squig* locus (especially among phenotypically wild type mice) we used a standard PCR assay that employed three primers: a single forward primer (F1, 5’-GTTACCAGGAGGTGCTCAAA-3’) that annealed 5’ to the *Meox1* deletion and two reverse primers—one that annealed within the *Meox1* deletion (R1, 5’-GTGAAATGTGAGAGAGGAGAGG-3') and one that annealed 3’ to the deletion, within *Gm11551, Exon 2* (R2, 5’-CCAGATCCCAGCAATCAAGATA-3'). Primers F1 and R1 direct the amplification of a 268 bp product specific to wild type BALB/cJ templates; the F1, R2 primer pair direct the amplification of a 456 bp product specific to *squig* templates.

## Results

**Fig. 1:**
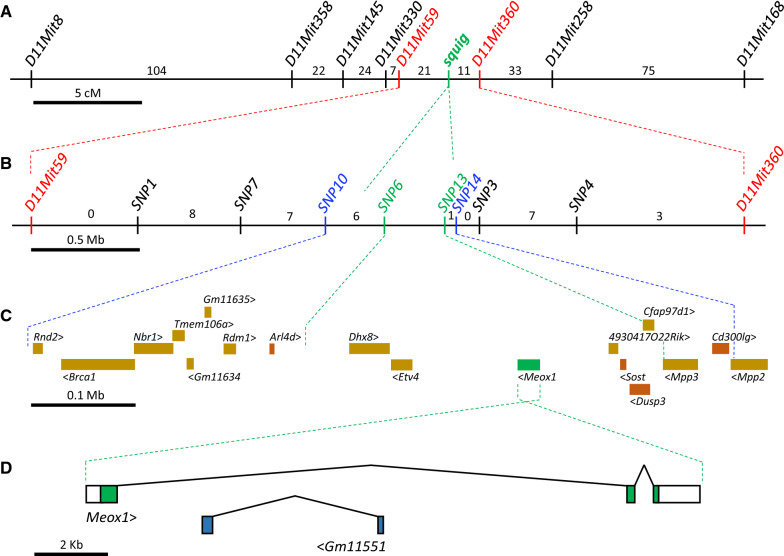
Genetic and physical maps of the *squig* region on distal mouse Chr 11. **A** Low-resolution genetic map of *squig* and eight microsatellite markers, based on the backcross panel described in Additional file 1 (Figure S2). The number of crossovers (out of 1008 meioses) located in each marker-defined interval is shown. As described in Figure S2, the *squig* mutation must be located within the 3.17 cM interval between markers *D11Mit59* and *D9Mit360.* **B** The *D11Mit59* to *D11Mit360* region is expanded to show the relative positions of 8 SNP markers used to type the 32 panel members recombinant in that 3.24 Mb interval. The number of crossovers located in each marker/SNP-defined interval is shown. The *squig* mutation must lie between *SNP10* and *SNP14*, and was never separated from *SNP6* nor *SNP13.* **C** The 0.6 Mb span between *SNP10* and *SNP14* includes 18 protein coding genes (shown as colored boxes). At least 5 of these (*Arl4d*, *Meox1*, *Sost*, *Dusp3* and *Cd300lg;* shown as orange or green boxes) are known to be expressed in the axial skeleton [2]; only *Meox1* (green box) is known to affect tail morphology when disrupted in mice [8, 9]. **D** The *Meox1* gene is expanded to show the 3 exons it comprises (with a 2 kb scale bar). Green boxes represent coding regions; white boxes indicate the 5’ and 3’ untranslated regions. *Meox1*, *Intron 1–2* includes a two-exon lncRNA (*Gm11551*, shown as blue boxes) that is transcribed from the complementary strand

To genetically map the *squig* mutation, F_1_ heterozygotes (made by crossing BALB/c-*squig*/*squig* mice with standard C57BL/6 J mice) were crossed with *squig*/*squig* mutants, producing 1008 backcross (N_2_) offspring that segregated for alternative alleles of *squig* and numerous molecular markers. Guided by Karst’s previous mapping efforts [1], DNAs isolated from this N_2_ panel were typed for eight, PCR-scorable microsatellite (dinucleotide repeat) markers [4] known to map to distal Chr 11. Additional file 4: Figure S2 shows the string of markers transmitted by the F_1_ parent to each of these 1008 N_2_ progeny. This haplotype analysis suggested that the *squig* gene must be located within the 3.2 cM region between *D11Mit59* and *D11Mit360* (Fig. 1A). This genomic interval includes *Rpl27* (for ribosomal protein L27), and, because defects in the related genes *Rpl24* and *Rpl38* have been shown to cause tail abnormalities in mice [5, 6] respectively, we investigated *Rpl27* as the potential basis of *squig*. However, DNA sequence analysis of all exons of *Rpl27* in *squig* mutants (data not shown) revealed no defects compared to wild type.

DNA samples from the 32 mice identified as having a crossover between *D11Mit59* and *D11Mit360* were typed next for eight SNPs known to lie in that 3.24 Mb interval. These eight SNP markers are described in detail in Additional file 2: Table S1 and Additional file 3: Table S2 and are designated herein as *SNP#*. This analysis located six crossovers that fell centromeric to *squig* (between *SNP10* and *SNP6*), and one crossover that fell distal to *squig* (between *SNP13* and *SNP14*) (see Fig. 1B), thus restricting the location of *squig* between *SNP10* and *SNP14* (and very near *SNP6* and *SNP13*, which were not meiotically separated from each other or from *squig*).

**Fig. 2:**
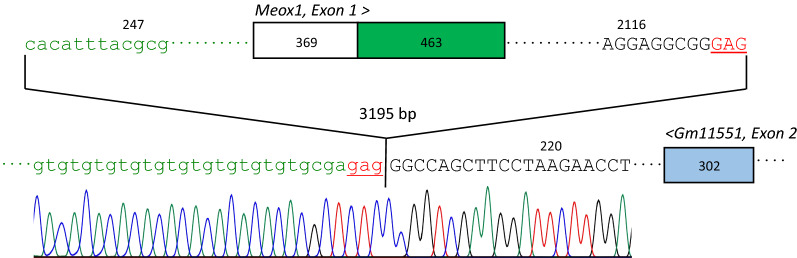
3195 bp including *Meox1*, *Exon 1* are deleted in the squiggle tail variant. Bases shown in lower-case green are from the *Meox1* promoter region, uppercase black letters are from *Meox1*, *Intron 1–2*. The upper boxed region represents *Meox1*, *Exon 1;* the 5’UTR is shown in white, the coding region is shown in green. *Exon 2* of the lncRNA-encoding gene known as *Gm11551*, which lies entirely within *Intron 1–2* of *Meox1*, is represented by a blue box (also see Fig. 1D). The number of base pairs that compose each segment is shown. A three-base direct repeat (5’-GAG-3’) found at the deletion breakpoint, a typical characteristic of spontaneous deletions in mammals [11], is underlined

The 0.6 Mb span from *SNP10* to *SNP14* includes 18 expressed genes (Fig. 1C) (which, incidentally, do not include *Rpl27*)*.* While four of these genes (*Arl4d*, *Sost*, *Dusp3* and *Cd300lg*) have been associated with abnormal bone morphology or mineralization [2] the homeodomain-containing transcription factor gene, *Meox1* (for mesenchyme homeobox 1), became our primary gene candidate due to its well-established role in axial skeleton formation [7–10]. DNA sequence analysis of all *Meox1* exons in wild type BALB/cJ and BALB/c-*squig/squig* genomic DNA revealed a 3195 bp deletion that extends from the *Meox1* promoter region to include all of *Exon 1* and part of *Intron 1–2* (Fig. 2). The deletion does *not* extend as far as predicted gene *Gm11551* [12] which encodes a lncRNA that is entirely contained within *Intron1-2* of *Meox1* and is transcribed from the complementary DNA strand (see Fig. 1D).

Next, we used a “3-primer” PCR test (see Additional file 5: Figure S3A) to rapidly screen for the presence of the *squig*-associated deletion of *Meox1, Exon 1* among 28 standard mouse strains but found no *Meox1, Exon 1* defect in any of them (see Additional file 5: Figures S3B and Additional file 6: S4), suggesting that the 3195 bp deletion is specific to the *squig* mutation.

## Discussion

Because *Meox1* and *squig* map to the same small region on Chr 11, because *squig* mutants display a severe, specific defect in *Meox1*, and because engineered mutations in *Meox1* produce similar recessive vertebral anomalies [including hemivertebrae, tail kinks and craniovertebral fusions, see [8, 9]] we suggest that *squig* is a spontaneous mutant allele of *Meox1* and recommend that its official designation be changed to *Meox1*^*squig*^.

At least four independent defects in the human MEOX1 orthologue [13–15] have been associated with Klippel-Feil syndrome-2 (KFS2), an autosomal recessive condition characterized by a short neck, low occipital hairline and reduced bilateral neck movements resulting from the fusion of cervical vertebrae [16]. Especially because the previously-described mouse variants [8, 9] are no longer extant, we suggest that the *Meox1*^*squig*^ variant described herein (available from The Jackson Laboratory as JAX stock #026620) could provide a highly relevant animal model for this inherited human disorder.

## Limitations

Rigorous proof for this gene assignment would require rescue of the mutant phenotype by the single addition of a wild type *Meox1* allele, or complementation testing with an extant, engineered *Meox1* mutation, for example. These formal tests were not performed here. The location of the antisense lncRNA *Gm11551* within the first intron of mouse *Meox1* may suggest a *cis* regulatory relationship [17] that warrants further investigation, although it is notable that the human MEOX1 orthologue does not harbor a similar lncRNA gene. While the *Meox1*^*squig*^ deletion does not disrupt the *Gm11551* coding sequence, we did not verify the normal expression or processing of *Gm11551* RNA.

## Supplementary Information

**Additional file 1: Figure S1.** A wild type heterozygote (left) and a mutant *squig/squig* mouse (right) at 10 days of age.

**Additional file 2: Table S1.** Description of SNP markers referred to in the Girard et al*.* (2022) text.

**Additional file 3: Table S2.** Location of SNP markers referred to in the Girard et al. (2022) text.

**Additional file 4: Figure S2.** Segregation of alleles of *squig* and eight microsatellite markers among 1008 intraspecific backcross progeny.

**Additional file 5: Figure S3.** A deletion including *Meox1, Exon 1* appears to be specific to the *squig* mutation.

**Additional file 6: Figure S4.** Original, uncropped photographs that were used to produce Figure S3B (as required by the editor).

## Data Availability

All data supporting the results of this article are included in this article and its additional files. Any materials or databases generated in this study are available from the corresponding author upon reasonable request.

## Abbreviations

Chr: Chromosome
lncRNA: Long, non-coding RNA
SNP: Single nucleotide polymorphism
KFS2: Klippel-Feil syndrome 2
*Meox1*: Mesenchyme homeobox 1
*Rpl27*: Ribosomal protein L27
