# Supplementary figures and images for: The squiggle tail (squig) mutation in mice is associated with a deletion in the mesenchyme homeobox 1 (Meox1) gene
Source: BMC Res Notes. 2022 Sep 23;15:305. doi: 10.1186/s13104-022-06192-z (PMC9502874; doi:10.1186/s13104-022-06192-z)

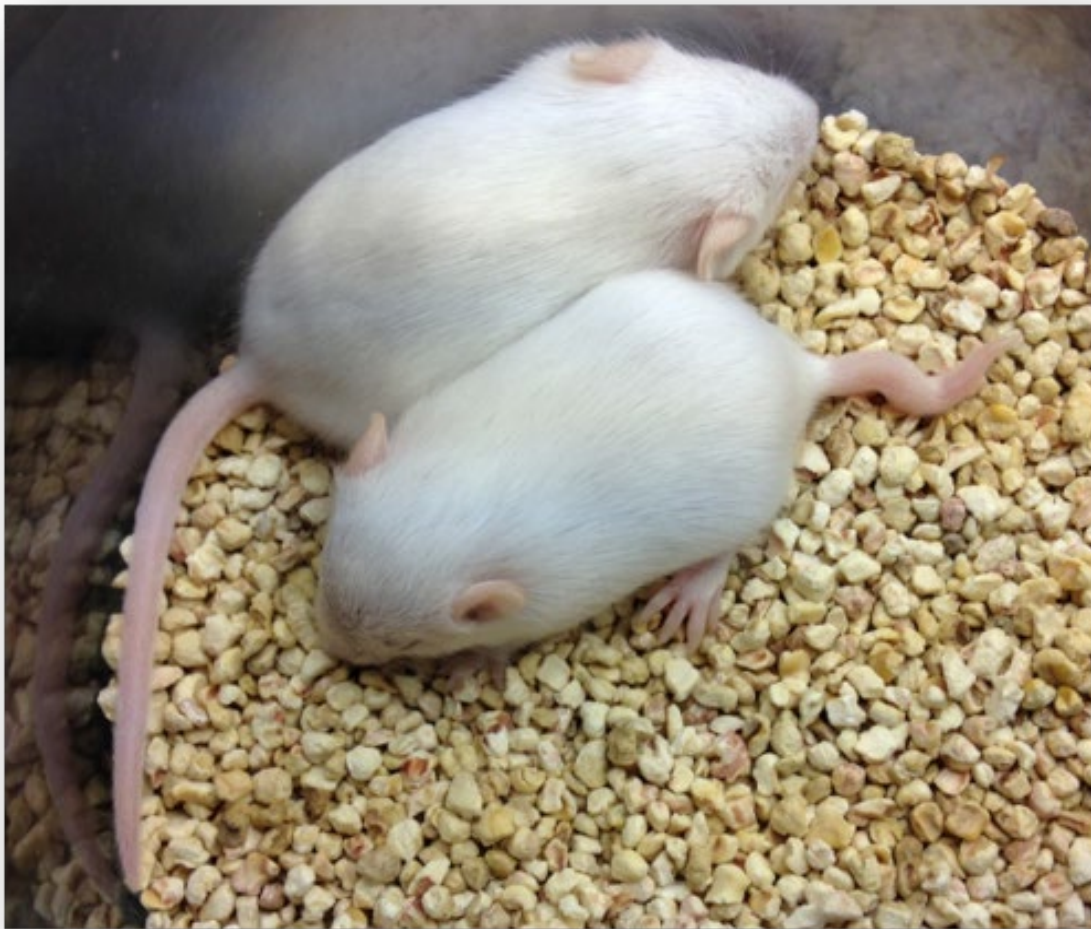

**Figure S1.** A wild type heterozygote (left) and a mutant *squig/squig* mouse (right) at 10 days of age.

Supplement: Supplementary file 1 — Additional file 1: Figure S1. A wild type heterozygote (left) and a mutant squig/squig mouse (right) at 10 days of age. [file 13104_2022_6192_MOESM1_ESM.pdf]
