# Supplementary material for: The squiggle tail (squig) mutation in mice is associated with a deletion in the mesenchyme homeobox 1 (Meox1) gene
Source: BMC Res Notes. 2022 Sep 23;15:305. doi: 10.1186/s13104-022-06192-z (PMC9502874; doi:10.1186/s13104-022-06192-z)
Supplement: Supplementary file 2 — Additional file 2: Table S1. Description of SNP markers referred to in the Girard et al. (2022) text. [file 13104_2022_6192_MOESM2_ESM.pdf]

Table S1. Description of SNP markers referred to in the Girard *et al.* (2022) text.

Designation in Girard *et al.* 2022	Official Designation	Forward Primer (5'-3')	Reverse Primer (5'-3')	Amplimer Length (bp)	Alleles (B/C=S)	Amplimer Sequence
SNP1	*rs3154913*	gggctctgttaaggaagtcg	tggtagagtcatggaggagac	361	G/A	5′ gggctctgtt aaggaagtcg TCATTGTCGC<br>CGCGACGCCT CCCCCACTCT GCAGCCGACC TCTGGGGTTC<br>CTACCCTTCC CCCCGCCCCC GCCCGGGAAC CCGGCCTCCA<br>CGATGAAGTC CACCTTCCTC CCCGCGGCAG AACAAGACCA<br>TGCCTCAAGC TCCCTGCTAG GCGGAAGCAG AAGGGGACAG<br>CAGACCCCCT AAGTGTACCC CTTCAGAGGG ATCTC**R**GTCG<br>ACTCCAGCTC AGATGGGGGC GGGGGGTGTC TGCAGGAGAG<br>CCATCGTCTC TGCATCTGGG GGCTGCCTCG CTGGCGGGTC<br>CACCTGCCCC AAGCCACGAG CTGCAAGCTC gtctcctcca<br>tgactctacc a 3′
SNP7	*rs27089317*	taccgatctagctgctactacc	tctgctggcaatctcaagg	162	A/G	5′ taccgatcta gctgctacta ccTATAGAGC<br>TTGTTACTTG TAGAGCAACC CATCCCTTGA TCAACTAAGG<br>T**R**GTTACAAA CAGCACCCTT TGGTGCTTTG ACCCACACCA<br>GGCTGGGGCA AGCCTTCCAA GGACCCAACT CGGccttgag<br>attgccagca ga 3′
SNP10	*rs29478873*	ggaacaggccctttcttatgg	atgtctgtggatggaaagatgg	243	T/C	5′ catcactttc attgtgcctt agCTCCTTTT<br>GGGGCCCAGG TTCAAATTCC ACAATTGAGA ACTGGAGGGA<br>CAGAGACTGG ACAGGAGATG GTGTGTGGGG GTGAGGGCAG<br>GCTGCTAAGA ATGGCAGGCT GGACCTGGTG TCTAAATGTT<br>ACCACGGCAA CTATGCTGCA GAGGCCTC**Y**G GGCTCCACAG<br>GCCAGGCAGT CTGGTGTTGC TATGACAACA GAccagatag<br>gctacagaga ggg 3′
SNP6	*rs584646540*	tttaaggccagcttggtctac	tggtgcatctgaagacaactaca	142	T/G	5′ tttaaggcca gcttggtcta cAGAGTGTTT<br>CTGAACAGCC GGGGCTACTC AGAAGAACCA TGTTTTTTTT<br>TTT**K**TTTTTT TTTTTTTTAA GATTTATTTA TTATTATATC<br>TAAGTACAct gtagttgtct tcagatgcac ca 3′
SNP13	*rs270211140*	cagctatgacctgtcccaatc	tggctcactagtccagtctataa	353	A/G	5′ cagctatgac ctgtcccaat cCCTGGGCAA<br>AGTGCTTCTG GACAGAGACA ACACGACAAC CCACACTTTG<br>AGTCTATACA CTTTTGACAG GACTGATTTA TTGTGGCTTA<br>GACAGAGCTT TGACAGATGG CAGTCATTTG ACTCCCACAA<br>TCCAGCACAC CAGGGGCCCC AGTCTGAAAA TAGAGACAGC<br>TTGGAACCGA GGCAGTAAAG AGACAGCTGC AC**R**CAAAGGA<br>CAGATCCATT CACATCAAGG ACTCACTTTC AGCATGGGGG<br>TCTTCAGAAA GGGGACAGGC AGCCTGACAT TCCTCAAGAG<br>TCCCCAAAGG CACTGCTGCA ttatagactg gactagtgag<br>cca 3′

SNP14	*rs45853985,*<br>*rs50914516,*<br>*rs45976113,*<br>*rs45871048,*<br>*rs238449189,*<br>*rs49319721,*<br>*rs49372367*	aagggaagatgggagagtaga	tggtgctggtctgtgtttc	219	C/T,<br>A/G,<br>A/G,<br>C/A,<br>-/TGCATA<br>C/T,<br>A/C	5′ aagggaagat gggagagtag agGAAGATTC<br>AGGCTCTGGA GTCTGGAATG GGGGGTTGTA AGAAATGGTG<br>GAGTTGAGGG GAACCYATAA GGRAACCTCA AAGAGCCACC<br>TCCAAGAGAA GGCAATCCTC TGACCCCCCT CRAAMTCAAC<br>CAAATGTGTG CATGTGCGAC ACAYACACAC ACACACACAC<br>MCCAAGGCCA gaaacacaga ccagcacca 3′
SNP3	*rs27009080*	caaccgactctggacataacc	agttcggccaaagacagc	255	G/A	5′ caaccgactc tggacataac cAGACCTGGA<br>CTCCAAGCTG AAAACCGATC CTGTGTCATC TAAATGRAGA<br>CTCAAGTGTG AGATGGGGAT AATGGCTTTC CACCTCATCA<br>CTGCTGGGAG ACTTAAATGT AGCAAAGGTG GTTTGGCAGA<br>TAATAAGGGT CCCATAAAAG GGTGTGATAC AGACCCCAGA<br>GAGAATTCTC TGTCTTTgct gtctttggcc gaact 3′
SNP4	*rs3681749,*<br>*rs4137585*	aaatggctagttcggttccc	attctacctcagcatcttctgc	442	T/C,<br>C/T	5′ aaatggctag ttcggttccc ATCACCCACA<br>TGGTAGCTCA CAAGCAGTTC CAAGGGATCC AGTACTCTCT<br>ACTGGACTCT GAGGGCTCCA GGCAYTCATG AAGTACACTT<br>ACATACATGC AATACTCATA TACATAAAAT GTAAATGAAT<br>TTTTTTTTAG CAAAGTAGCT TGGCATGGTG GCACTTTTAA<br>ACCTACCACT CAGGAGGCAG GGGCAGACCT GGTCAACGTA<br>CAGTCAGTGC TACACAGTAA GATCCTGTCT AAAACCAAAA<br>CAAAGATGAA AACCAATAAA AAATTATATA TATATATATA<br>TATATATATA TATATATATA TATATATATA TATATACAYA<br>CACACACACA CACACATACA TACATACATA TATGTAGGGC<br>ATCGTGCGGT ACTTATACAG CAGTATATGT gcagaagatg<br>ctgaggtaga at 3′

SNP markers are listed in order of their positions on Chr 11 (see Table S2). Primers listed (in lower case) were used to amplify genomic DNA from strains C57BL/6J (abbreviated B), BALB/cJ (abbreviated C) and BALB/c-squig/J (abbreviated S). Amplimers were sequenced by primer extension (Keck Foundation Resource Laboratory at Yale University, New Haven, CT). Nucleotides that differ between two strains are shown in red, where S = G or C, Y = C or T, K = G or T and R = A or G. Official designations from dbSNP release 150. These data accessed through the Mouse Genome Database (MGD) at the Mouse Genome Informatics website, The Jackson Laboratory, Bar Harbor Maine, http://www.informatics.jax.org (March 2022)(ref. 2) and the Ensembl Mouse Genome Browser (EMGB), http://ensembl.org/Mus_musculus, Release105.39 (Dec 2021)(ref. 3).
