# Supplementary material for: The squiggle tail (squig) mutation in mice is associated with a deletion in the mesenchyme homeobox 1 (Meox1) gene
Source: BMC Res Notes. 2022 Sep 23;15:305. doi: 10.1186/s13104-022-06192-z (PMC9502874; doi:10.1186/s13104-022-06192-z)
Supplement: Supplementary file 3 — Additional file 3: Table S2. Location of SNP markers referred to in the Girard et al. (2022) text. [file 13104_2022_6192_MOESM3_ESM.pdf]

Table S2. Location of SNP markers referred to in the Girard et al. (2022) text.

Designation in Girard *et al*. 2022	Official Designation	Position (GRCm Build 39)	5' Flanking Gene	3' Flanking Gene
SNP1	*rs3154913*	11:100510205	*Dnajc7*: Intron	*Nkiras2*: 5'UTR
SNP7	*rs27089317*	11:100969059	*Naglu*	*Hsd17b1*
SNP10	*rs29478873*	11:101353607	*Vat1*: Intron	*Vat1*: Intron
SNP6	*rs584646540*	11:101603325	*Arl4d*	*Dhx8*
SNP13	*rs270211140*	11:101890634	*Mpp3*: 3' UTR	*Mpp3*: 3' UTR
SNP14	*rs45853985, rs50914516, rs45976113, rs45871048, rs238449189, rs49319721, rs49372367*	11:101949028	*Mpp2*: 3' UTR	*Mpp2*: 3' UTR
SNP3	*rs27009080*	11:102048079	*Tmem101*	*Lsm12*
SNP4	*rs3681749, rs4137585*	11:102489885, 11:102490139	*Gm11627*	*Fzd2*

SNP markers are listed in order of their positions on Chr 11. Official designations and base-pair positions on mouse Chr 11 are from GRCm39. These data were accessed through the Ensembl Mouse Genome Browser (EMGB), http://ensembl.org/Mus_musculus, Release 105.39 (Dec 2021)(ref. 3).
