# Supplementary material for: The squiggle tail (squig) mutation in mice is associated with a deletion in the mesenchyme homeobox 1 (Meox1) gene
Source: BMC Res Notes. 2022 Sep 23;15:305. doi: 10.1186/s13104-022-06192-z (PMC9502874; doi:10.1186/s13104-022-06192-z)
Supplement: Supplementary file 4 — Additional file 4: Figure S2. Segregation of alleles of squig and eight microsatellite markers among 1008 intraspecific backcross progeny. [file 13104_2022_6192_MOESM4_ESM.pdf]

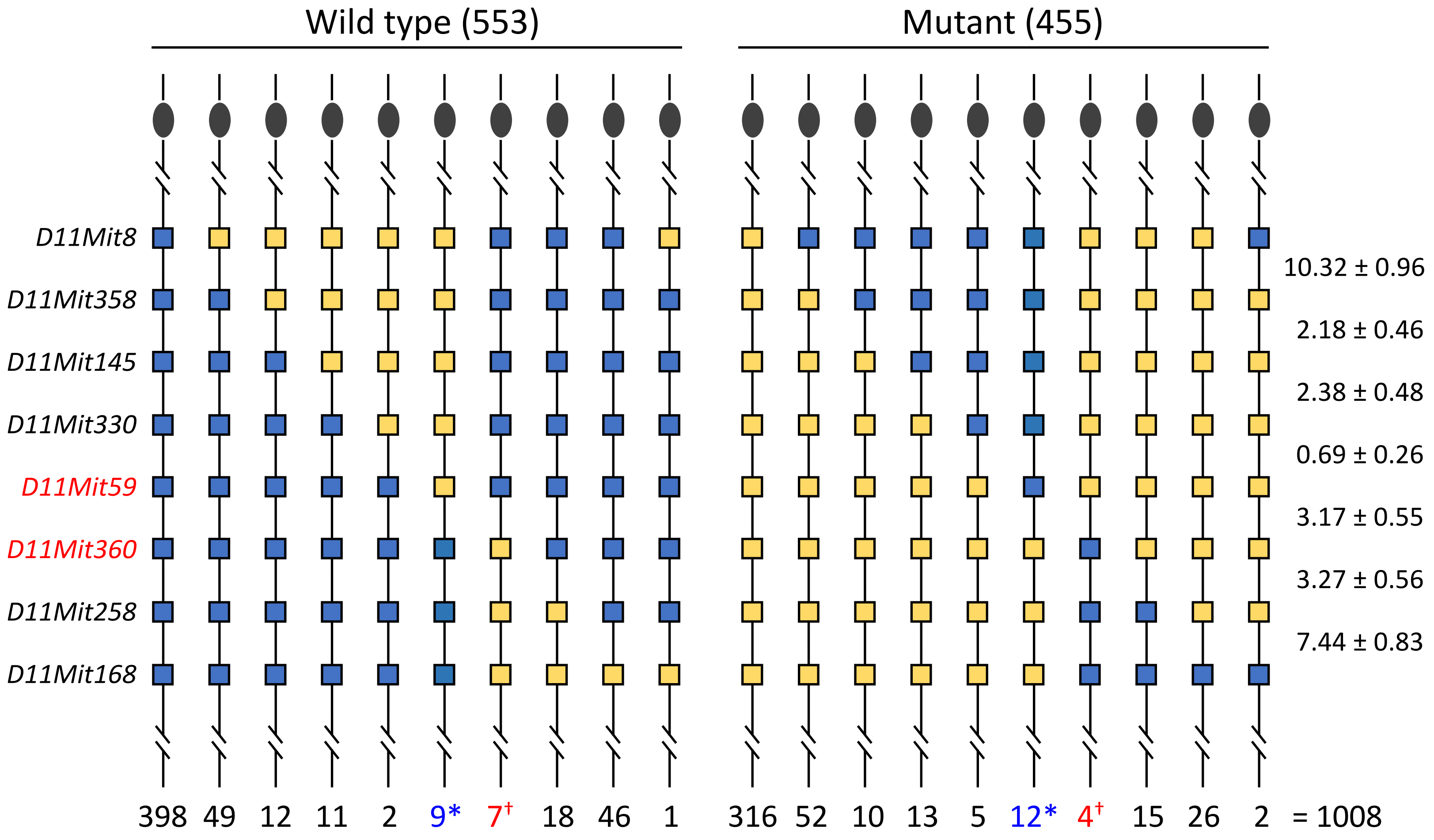

Figure S2. Segregation of alleles of *squig* and eight microsatellite markers among 1008 intraspecific backcross progeny. Heterozygous (BALB/c-*squig*/J x C57BL/6J)F_1 mice were backcrossed to homozygous BALB/c-*squig*/J mutants. The resulting progeny were scored for tail phenotype and a DNA sample from each mouse was typed for the microsatellite markers named at the left. Only the Chr 11 haplotype transmitted by the F_1 parent is shown. A knob at the top of the haplotype represents the centromere, and the number of mice inheriting that haplotype is shown below it. Blue boxes represent C57BL/6J-derived alleles; yellow boxes represent BALB/c-*squig*-derived alleles. The number of mutant progeny recovered was significantly less than the 50% expected from a testcross ($X^2 = 9.53$; $P < 0.002$), suggesting that mutants may be only about 82% viable (455 mutants scored / 553 mutants expected, assuming wild type is fully viable), at least by weaning age. The 9 wild type and 12 recombinant mutants marked with a blue asterisk show that the *squig* locus must lie distal (telomeric) to *D11Mit59*. The 7 wild type and 4 mutant recombinants marked with a red dagger show that *squig* must lie centromeric to *D11Mit360*. Genetic distances (in percentage recombination) are shown to the right (± 1 Standard Error).
