# Supplementary material for: The squiggle tail (squig) mutation in mice is associated with a deletion in the mesenchyme homeobox 1 (Meox1) gene
Source: BMC Res Notes. 2022 Sep 23;15:305. doi: 10.1186/s13104-022-06192-z (PMC9502874; doi:10.1186/s13104-022-06192-z)
Supplement: Supplementary file 5 — Additional file 5: Figure S3. A deletion including Meox1, Exon 1 appears to be specific to the squig mutation. [file 13104_2022_6192_MOESM5_ESM.pdf]

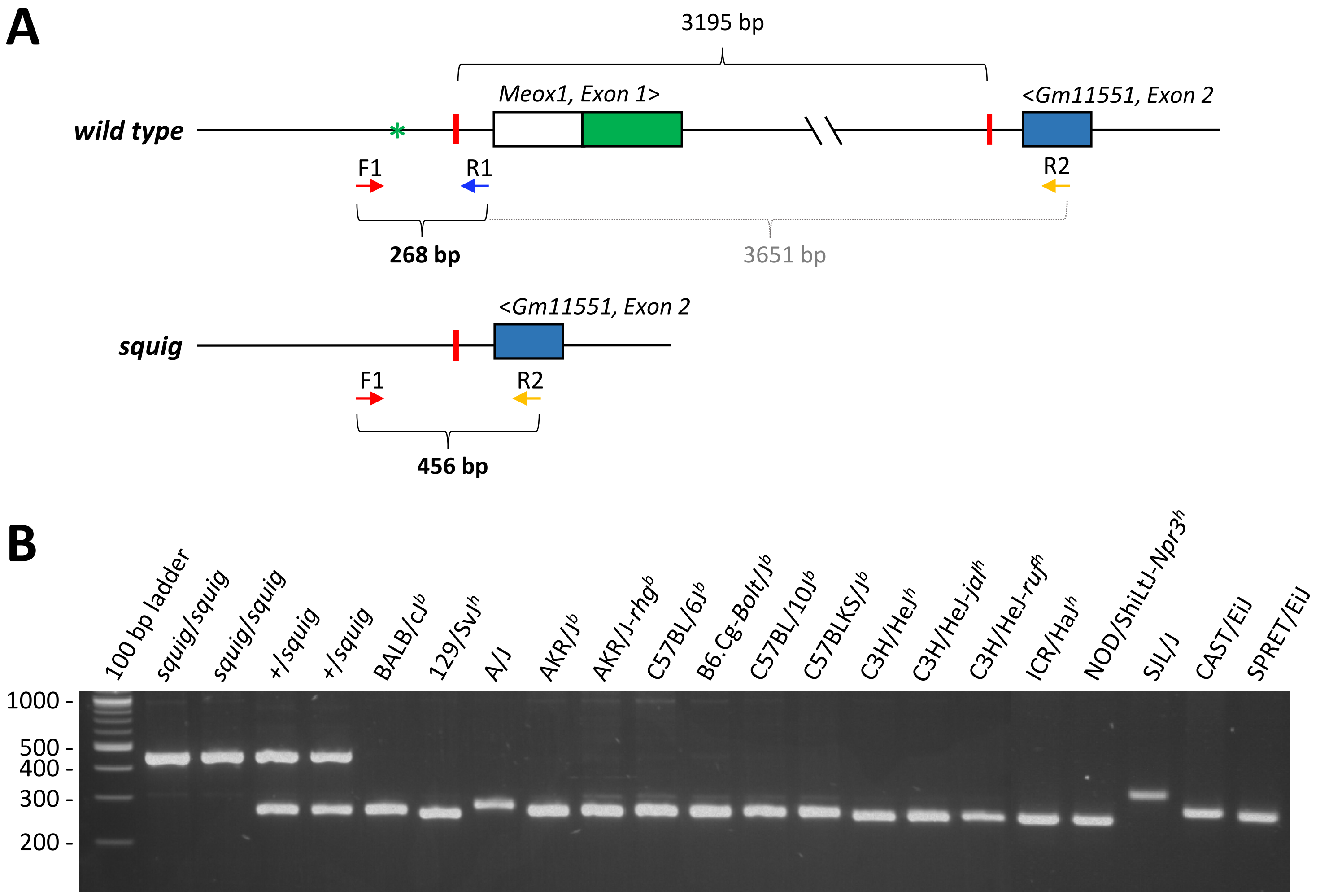

Figure S3. A deletion including *Meox1, Exon 1* appears to be specific to the *squig* mutation. **A** The rationale of a 3-primer PCR test for rapid detection of the 3195 bp deletion described in Fig. 2. The positions and orientation of the 3 primers, named F1, R1 and R2, are shown by arrows below the region that includes *Meox1, Exon 1* and *Gm11551, Exon 2*. (Primer sequences are described in the Methods section.) The *squig*-associated deletion is bounded by a 3-base direct repeat (represented by red lines here and see Fig. 2). Primers F1 and R1 direct production of a 268 bp amplimer based on BALB/c-derived wild type templates, while primers F1 and R2 direct production of a 456 bp amplimer based on *squig* templates. **B** Typical results of deletion screening among various inbred mouse strains. PCR-generated amplimers based on genomic DNA templates isolated from the mouse strains indicated were electrophoresed through 3.5% NuSieve® agarose. Only reactions using *squig/squig* or *+/squig* templates yielded the mutation-specific 456 bp amplimer. Wild type amplimers (generated by primers F1 and R1) are variable in length depending on the number of dinucleotide (5’-gt-3’) repeats present in the *Meox1* promoter region (just upstream of the *squig*-associated deletion—designated by a green asterisk in Part A and see Fig. 2) and/or other insertions and deletions (as have been previously reported [3]) for some of these standard inbred strains. For example, strains marked *b* (including BALB/cJ) gave a 268 bp amplimer, strains marked *h* yielded a 260 bp amplimer, A/J templates yielded a 278 bp amplimer, SJL/J templates generated a 314 bp amplimer, CAST/Ei gave a 280 bp amplimer, and SPRET/Ei templates yielded a 273 bp amplimer (these lengths were verified by primer-extension sequence analysis).
