# Supplementary material for: The squiggle tail (squig) mutation in mice is associated with a deletion in the mesenchyme homeobox 1 (Meox1) gene
Source: BMC Res Notes. 2022 Sep 23;15:305. doi: 10.1186/s13104-022-06192-z (PMC9502874; doi:10.1186/s13104-022-06192-z)
Supplement: Supplementary file 6 — Additional file 6: Figure S4. Original, uncropped photographs that were used to produce Figure S3B (as required by the editor). [file 13104_2022_6192_MOESM6_ESM.pdf]

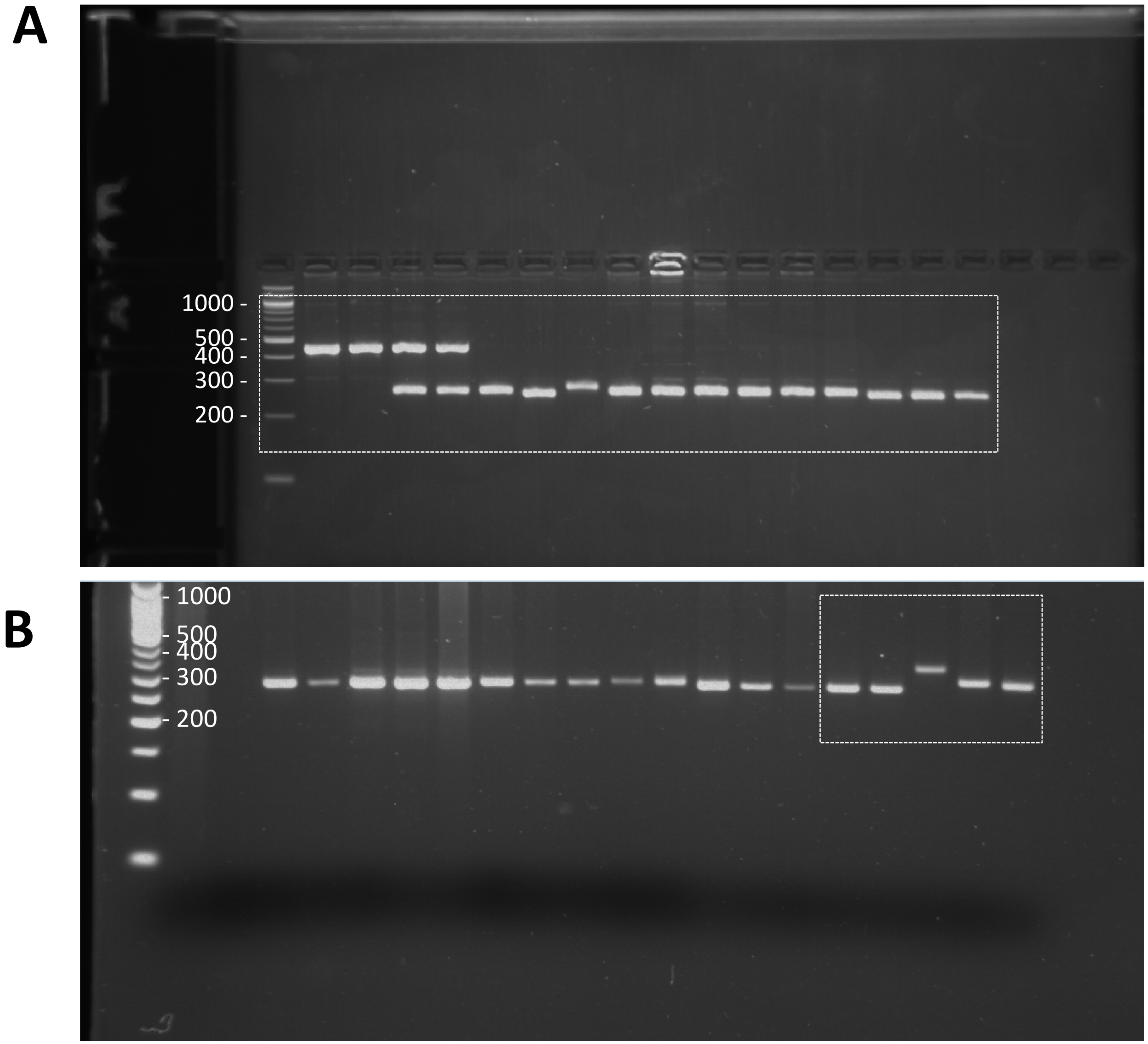

Figure S4. Original, uncropped photographs that were used to produce Figure S3B (as required by the editor). Cropped areas used in Figure S3B are indicated by dotted white boxes. The source of template DNA used in each PCR is shown above each corresponding lane in Figure S3B. In panel A, a 100 bp ladder was used as a size standard (left-most lane). In panel B, a 50 bp ladder was used instead. Precise amplimer sizes (described in the Figure S3 legend) were verified by primer-extension sequence analysis.
